# *Salmonella* Oranienburg haemorrhagic cystitis in an immunocompetent young male

**DOI:** 10.1099/jmmcr.0.005105

**Published:** 2017-08-08

**Authors:** Jiasian Teh, Mark Quinlan, Damien Bolton

**Affiliations:** Department of Urology, Austin Hospital, Heidelberg, Melbourne, Victoria 3084, Australia

**Keywords:** Salmonella oranienburg, Haemorrhagic cystitis, IV antibiotics

## Abstract

**Introduction.** Non-typhoidal salmonella (NTS) bacteriuria is extremely rare. Haemorrhagic cystitis is defined by urinary symptoms including haematuria, frequency, urgency and dysuria. Reports of haemorrhagic cystitis caused by NTS are exceptionally uncommon, especially in immunocompetent, young, male patients.

**Case presentation.** A 27-year-old male with no past medical history presented to the Emergency Department with a 24 h history of visible haematuria having returned five days earlier from a five month trip across South America. He also reported one week of suprapubic pain with associated dysuria, frequency, difficulty voiding and fevers. A non-contrast abdominal and pelvic CT scan showed a grossly thick-walled bladder with marked peri-vesical stranding, strongly suggestive of cystitis, with an unremarkable appearance of the remainder of the urinary tract. Urine culture at presentation subsequently grew *Salmonella* Oranienburg. The patient reported total symptomatic relief following just one week of oral antibiotics with no recurrence to date.

**Conclusion.** NTS urinary tract infection (UTI), especially in healthy young people, is very rare. In such cases, the existence of underlying diseases must be considered, especially diabetes mellitus, urological abnormalities and immunosuppression. However, a diagnosis of NTS UTI should also be among the differentials in those presenting with acute urinary symptoms preceded by gastrointestinal upset, especially following travel in underdeveloped countries. Antibiotic therapy is invariably indicated and close follow-up is warranted due to the risk of several potentially serious complications.

## Abbreviations

CT, computed tomography; IV, intravenous; NTS, non-typhoidal salmonella; STI, sexually transmitted infection; UTI, urinary tract infection.

## Introduction

Salmonellosis is a major cause of morbidity with an estimated 800 000 to 4 million cases occurring annually in the USA [1]. Non-typhoidal salmonellosis is a food-borne infection of huge public health concern. Non-typhoidal Salmonella (NTS) is an enteroinvasive bacterium. NTS was first mentioned as a causative agent of urinary tract infection (UTI) in 1946 by Seligman *et al*. [2]. A large contemporary case series on the topic reported that NTS bacteriuria represented 0.07 % of all UTIs diagnosed in that particular area over a 16 year period (*n*=19) [3]. Furthermore, the vast majority of reported cases to date of NTS UTI have occurred in susceptible patients, namely those with diabetes mellitus, urolithiasis, other urological abnormalities or immunocompromised conditions, although this assertion is contested by some authors [4]. Rarer described risk factors include the handling of exotic pet reptiles, such as iguanas. Affected patients are typically middle aged or older.

Saphra *et al*., in a large review of 7779 NTS infections in adults, found just 49 cases (0.63 %) of UTI [5]. Furthermore, reports of haemorrhagic cystitis caused by NTS are exceptionally rare, especially in immunocompetent young patients. Haemorrhagic cystitis is defined by lower urinary tract symptoms that include haematuria, dysuria, frequency, urgency and suprapubic pain and is caused by viral or bacterial infection or chemotherapeutic agents.

We herein report a case of a healthy 27-year-old man, with no history of UTIs, whose clinical, radiological and microbiological investigations confirmed NTS haemorrhagic cystitis. He required hospitalisation for intravenous (IV) antibiotics and fluid resuscitation. He made a full recovery with no symptom recurrence to date.

## Case Report

A 27-year-old male with no past medical history whatsoever presented to the Emergency Department with a 24 h history of visible haematuria having returned five days earlier from a five month trip across South America. He also reported one week of suprapubic pain with associated dysuria, frequency, difficulty voiding and fevers. Two weeks prior to returning home from South America, he described a three day history of sweats, fevers, nausea, vomiting and abdominal cramps. He had a single episode of diarrhoea at the time. He had also experienced intermittent bouts of diarrhoea earlier on his trip. He denied any penile discharge or risk taking behaviour including IV drug use. He was sexually active with one stable, female partner. He had a negative sexually transmitted infection (STI) screen one year prior.

**Fig. 1.**
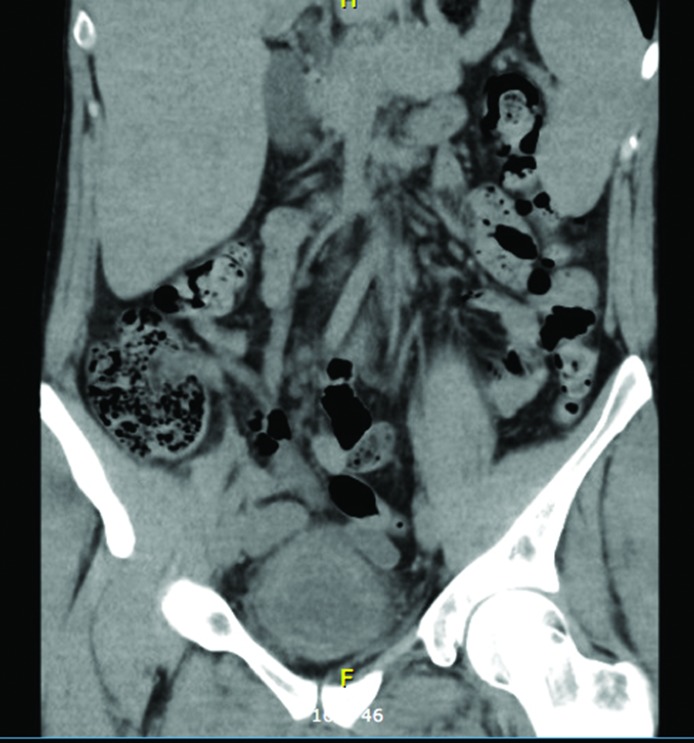
Coronal non-contrast CT KUB showing a markedly thick-walled bladder with peri-vesical stranding consistent with cystitis.

On examination, he was tachycardic with a heart rate of 120 b.p.m. His blood pressure was 106/72 mmHg, his temperature was 36.7 °C and his oxygen saturations were 95 % on room air with a respiration rate of 18 b.p.m. Lab findings were as follows: white blood cells (WBC) 9.6×10^9^ l^−1^, haemoglobin 133 g l^−1^, neutrophils 7.2×10^9^ l^−1^, creatinine 75 mmol l^−1^, C-reactive protein (CRP) 30.2 mg l^−1^. Two sets of blood cultures taken at the time of presentation were negative. Urinalysis revealed over 500×10^6^ leukocytes and erythrocytes l^−1^. He was resuscitated with IV fluids and initially treated with IV Ampicillin and Gentamicin. A non-contrast abdominal and pelvic CT scan (to rule out urolithiasis) showed a grossly thick-walled bladder with marked peri-vesical stranding strongly suggestive of cystitis (see Fig. 1), with an unremarkable appearance of the remainder of the urinary tract. Urine culture at presentation subsequently grew *Salmonella* Oranienburg. His symptoms settled quickly and after consultation with our microbiology service, he was discharged home after two days with a two week course of oral Amoxycillin–Clavulanate 875/125 mg BD.

He underwent a delayed, elective, outpatient flexible cystoscopy after repeat urine culture was sterile; this demonstrated multiple, diffuse, flat, erythematous areas presumed to be secondary to cystitis and which were not considered concerning. The rest of his lower urinary tract was completely normal at cystoscopy. A further follow-up flexible cystoscopy six weeks later showed complete resolution of these findings. The patient reported total symptomatic relief following just one week of oral antibiotics and he remained well once oral antibiotics were stopped after two weeks with no symptom recurrence to date (eight months post presentation at the time of writing).

## Discussion

Salmonellosis continues to occur in industrialised countries despite advances in individual and collective sanitation and careful monitoring of food processing. The main mode of transmission is from food products contaminated with animal products or waste, most commonly eggs and poultry, but also undercooked meat, unpasteurized dairy products, seafood and fresh produce. NTS causes infections that may have many different clinical presentations [6]. Gastroenteritis is the most common of these and it is usually a self-limiting illness. Less frequent but more severe presentations are bacteraemia and focal infections. In uncomplicated *Salmonella* gastroenteritis, antibiotic therapy is not recommended because it does not shorten the disease duration. However, in cases of sepsis or local extra-intestinal infection, prompt antibiotic administration is needed [7].

UTI from NTS is very rare, accounting for 0.01–0.07 % cases of UTIs in various studies [1, 3]. *Salmonella* Oranienburg is recognised as a food-borne pathogen which has been associated with outbreaks in several countries but which nonetheless remains a relatively uncommon serotype [8]. Infection of the urinary tract due to NTS usually occurs in immunocompromised individuals, including patients with malignancy, human immunodeficiency virus infection or diabetes mellitus and patients receiving corticosteroid therapy or treatment with other immunotherapeutic agents [9]. It can also be associated with structural abnormalities of the urinary tract including urolithiasis, chronic pyelonephritis, rectovesical fistula, urethrorectal fistula, hydrocoele and post-transurethral resection of the prostate (TURP) [1, 10]. Our patient did not have any of these predisposing factors. The modes of infection include direct urethral invasion by faecal flora or haematogenous spread from gastroenteritis [11]. It usually presents as typical symptoms of UTI. In a retrospective analysis of 799 isolates of NTS from urine, serotypes group C1 and E were most commonly associated with UTIs [12]. Given the organism involved in our case and the chronology of events, we believe it’s highly likely that he contracted this infection while in South America rather than upon his brief return to Australia. To the best of the patient’s knowledge, none of his contacts were similarly affected.

There are opinions suggesting that the relationship of NTS UTI with genitourinary abnormalities and immunosuppression is likely to be an overestimation as a result of bias and it is important to keep NTS in the differential of potential pathogens causing UTIs in patients without any overt predisposing factors [4]. That said, however, we believe an episode of NTS UTI, especially in males, should be considered as a potential surrogate marker of underlying predisposing factors, namely unrecognised immune system suppression or compromise of genitourinary anatomy.

NTS UTI may be difficult to treat, but early institution of antibiotics is associated with favourable outcome. Antibiotics with a high intracellular concentration should be used as *Salmonella* has the tendency to grow intracellularly [13]. Prolonged antibiotic treatment should be considered due to the high frequency of complicating conditions due to *Salmonella* UTI such as pyelonephritis, renal insufficiency, nephritic syndrome, urolithiasis, genitourinary abscess, recurrence and chronic bacteriuria but it should be noted that the infection can be recurrent despite prolonged antibiotic treatment [3, 13]. It seems likely that the organism in our case arose via a gastrointestinal rather than systemic route but without being certain of that, it may possibly represent further evidence of invasive NTS which has recently become the most significant *Salmonella*-related infection worldwide when mortality is considered. By way of illustrating this, invasive NTS disease was estimated to have caused 3.4 million illnesses and 681 316 deaths in 2010, with the most disease in Africa, placing it among some of the most deadly infectious diseases on the continent [14]. The predominant NTS strain causing invasive disease in Africa, *Salmonella* Typhimurium ST313, appears to show genomic features of differential host adaptation and convergent evolution with *Salmonella* Typhi [14]. Interestingly, In Africa, NTS strains appear to be different from those that cause diarrheal disease in industrialized countries, in that they more often cause invasive disease with bacteraemia [14].

In summary, NTS UTI, especially in healthy young people, is very uncommon. In such cases, the existence of underlying diseases must be considered, especially diabetes mellitus, urological abnormalities and immunosuppression. However, a diagnosis of NTS UTI should also be among the differentials in those presenting with acute urinary symptoms preceded by gastrointestinal upset, especially following travel in under-developed countries. Antibiotic therapy is invariably indicated and close follow-up is warranted due to the risk of several potentially serious complications.
